# Visor flap for total upper and lower lip reconstruction: a case report

**DOI:** 10.4076/1752-1947-3-7312

**Published:** 2009-06-09

**Authors:** Peter Nthumba, Louis Carter

**Affiliations:** 1 Department of Surgery, AIC Kijabe Hospital, Kijabe 00220, Kenya, Africa

## Abstract

**Introduction:**

Noma, aptly named the 'face of poverty', is a scourge with a mortality rate of up to 90% that affects some 140,000 people each year, predominantly children in the sub-Saharan 'noma belt'. Survivors of the acute attack suffer severe facial disfigurement from loss of facial tissue and scarring. Surgical reconstruction of noma defects is a major challenge, especially in Africa, where the majority of cases occur.

**Case presentation:**

We report the case of a 40-year-old Somali man who presented with severe facial disfigurement, including total absence of both upper and lower lips. After a failed initial reconstruction, a combination of platysma flaps and a left deltopectoral flap provided mucosal lining, while a scalp visor flap served to recreate upper and lower lips, the beard and moustache.

**Conclusion:**

The scalp visor flap offers a simple but extremely versatile tool for use in midfacial reconstruction, especially in the male, providing neo-lip tissue, a moustache and a beard. This is the first report of a simultaneous total upper and lower lip reconstruction using a scalp visor flap, in the English literature. We also emphasize on a process of transfer of skills to enable local surgeons to effectively manage the challenge that noma presents.

## Introduction

Noma, also known as cancrum oris, primarily affects orofacial structures. There is a strong correlation between noma and poverty, malnutrition, poor oral hygiene and infectious diseases [1] - conditions that are widespread in sub-Saharan Africa, particularly within strife-ridden communities. This scourge has been aptly called the 'face of poverty' [2], and afflicts mainly children in the developing world, with only a few cases reported from the developed world - these involve patients with debilitating diseases such as HIV/AIDS, diabetes mellitus and hematological disorders [3]-[4]. Noma was eradicated in developed countries because of improved standards of living, a better understanding of the disease and the use of antibiotics, while in other places, a significant fall in mortality was reported (from about 90% to 10%) [5]. Unfortunately, about 140,000 new cases of noma still occur each year in the sub-Saharan "noma belt". Ninety percent of these children die without any treatment [6].

Survivors of the acute stage suffer severe destruction of the midface, including lips, cheeks, maxilla and mandible, nose and occasionally, the orbit [5]. Progressive scarring, trismus, oral incontinence and mandibulo-maxillary synostosis leave the patient malnourished, severely disfigured and with speech difficulties.

Although cases of noma have been reported from East Africa (Kenya, Uganda and Tanzania) [7], it remains somewhat uncommon in this region. Political upheaval in surrounding countries over the years have created large populations of refugees, who have brought with them health problems hitherto unfamiliar to many physicians in the region; chief amongst them is noma, with its numerous management challenges.

This case report describes the authors' experience with the first noma patient managed at our hospital, and the valuable lessons learnt. This is also the first report of a total upper and lower lip, beard and moustache reconstruction using a scalp visor flap in the English literature.

## Case presentation

A 40-year-old Somali man presented to our hospital with a large midfacial defect, inability to open his mouth, oral incompetence and incoherent speech. Eight years prior to his presentation, he had noted a small blister in his left maxillary buccal mucosa, which had ruptured and a few weeks later had left a small ulcer that progressively increased in size with time. He eventually lost both his upper and lower lips, parts of the maxilla and some teeth, as well as the ability to open his mouth. Concurrently, he had developed difficulties with eating and speech.

**Figure 1:**
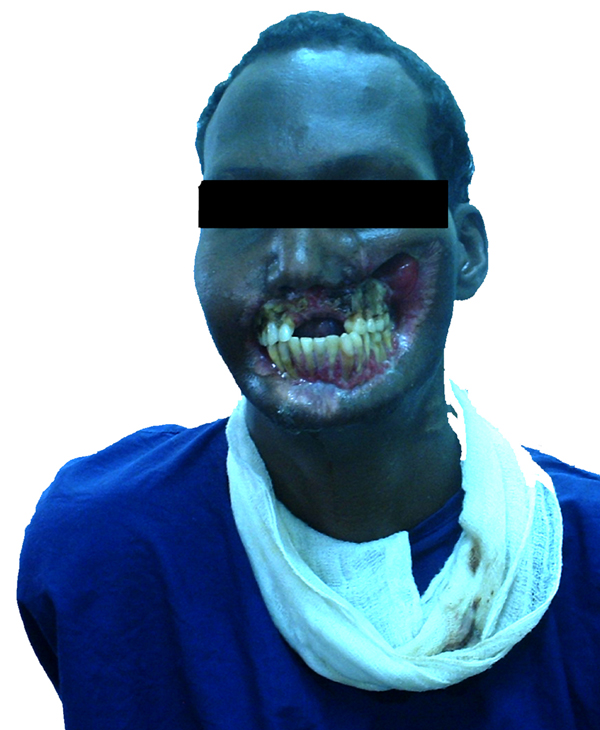
**Picture showing extent of defect, with complete absence of lip tissue, as well as inability to open mouth**.

On examination, he had a hideously deformed face - an extensive ulcer covered what used to be his mouth and midface. A deep ulcer cavity with a foul-smelling purulent discharge covered what used to be his left maxilla. Most of what remained of his left upper and lower dentition was visible through the soft tissue defect. On the right side, the esion was less extensive but also had no lip tissue (Figure 1). An orthopantogram revealed bilateral chronic osteomyelitis of his midfacial skeleton with bilateral maxilla-mandibular ankylosis. He did not have diabetes mellitus, HIV/AIDS, or any other co-morbidity.

A feeding gastrostomy and a tracheostomy preceded the midfacial reconstruction, which initially involved bilateral excision of the bony block between the mandible and maxillae, mobilization of the temporomandibular joint and soft tissue coverage achieved by use of bilateral pectoralis major muscle myocutaneous flaps.

Unfortunately, poor communication, misunderstanding and significant differences in surgeon and patient expectation led to the patient destroying this initial reconstruction on the first 3 postoperative days. He had expected to look his "normal old self" immediately postoperatively, and such was his disappointment with the results, that he stoically tore through his flaps, destroying the entire reconstruction.

**Figure 2:**
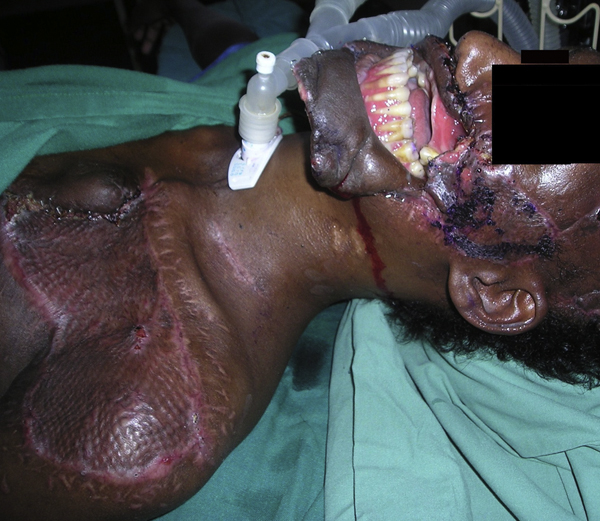
**The deltopectoral flap was raised, and fashioned to form the inner lining**. Chest defect at 6 weeks.

After discussion with the patient and his relatives, a staged reconstruction was agreed upon. Bilateral platysma flaps and a left deltopectoral flap were used for intra-oral lining, with the deltopectoral flap providing the lower lip lining, along with additional bulk needed to fill the defect over the left maxilla (Figures 2 and 4). A scalp visor flap was designed and prefabricated with a skin graft, and subsequently raised a week later. This was used to cover the midfacial defect, reconstruct his lips, and provide a moustache and beard, all as a single unit. The scalp defect was skin grafted.

**Figure 3:**
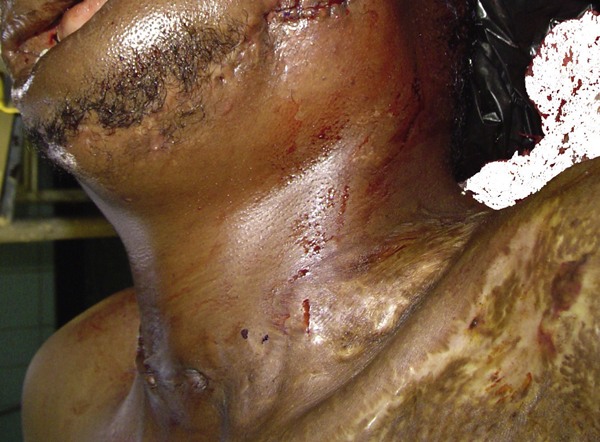
**Mature scars over neck and chest**. The platysma donor sites healed completely, with inconspicuous scars.

**Figure 4:**
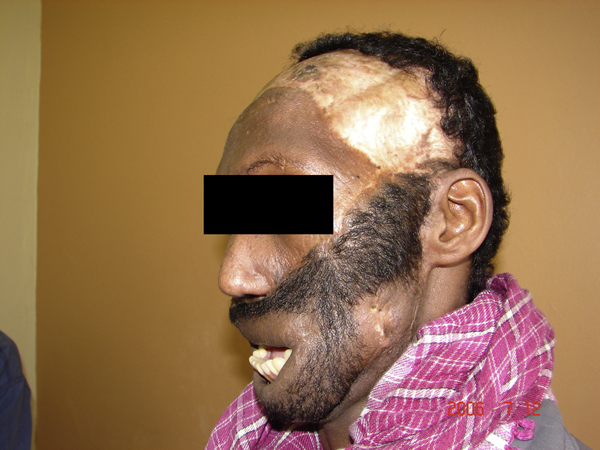
**Defect reconstructed, with ability to open mouth**. Note resulting scalp donor alopecia.

Although continence to solids was gained, he remained incontinent to liquids. Non-adherence to postoperative rehabilitation led to significant loss of mouth opening, from 35 mm immediately postoperatively to about 10 mm at 2 years.

## Discussion

Reconstruction in noma surgery is a surgical adventure not to be taken lightly; for this reason many workers have preferred transferring these patients to hospitals in the developed world, where supporting technology and skills make the surgeries much easier and safer to perform [8]. This strategy, although successfully running for many years, is a very expensive undertaking, when compared to the alternative where the patient is managed locally. Managing these patients locally offers multiple advantages including treatment within their own locale, a lower cost per surgery and therefore larger volumes of patients, and more importantly permits the transfer of skills and technology over time, through the training of local surgeons [9], as has also been our experience [10]. Sustained quality management of patients in the absence of the visiting specialist surgical team, is proof of successful transfer of knowledge, skills and technology. Continued partnership between the visiting specialist and local surgical team further enhances patient care by helping expand and maintain skills locally, while simultaneously freeing up available funding for the care of more patients as well as the purchase and/or maintenance of necessary equipment, a much cheaper, more effective and sustainable solution to the challenges facing reconstructive surgery in Africa and other developing environments.

In the presence of remaining lip tissue, the goals of lip reconstruction include the restoration of oral competence, maintenance of maximum oral aperture, mobility, sensation when possible, and cosmesis [10,11]. When there is absolutely no lip tissue available however, surgery can only attempt to reconstruct an alternative that will permit the acceptance and re-entry of the patient back into society. Form becomes paramount above function and beauty. While either free flaps or loco-regional flaps may be used in noma surgery, the type of reconstruction is largely determined by the surgeon's preference, skills, funding and technology.

Free flaps offer the reconstructive surgeon considerable versatility. Single stage reconstruction with appropriate tissue bulk and the avoidance of additional facial scars/incisions are important advantages. They are, however labor- and technology-dependent, expensive, and demand considerable technical skills. Free flaps are relatively new entrants in lip reconstruction. While most workers prefer the radial forearm flap use of the gracilis muscle and the anterolateral thigh flaps have also been reported [11]. Microvascular reconstruction has been successfully performed in sub-optimal environments, proving that these skills and technology can be modified to suit the operating theater conditions found in most developing countries [12]. This service will be a significant improvement on the quality of care locally available to these patients.

Loco-regional flaps provide excellent color match, minimize the number of surgical procedures, and are generally fail-safe, requiring simple postoperative care.

The scalp visor flap has an excellent blood supply, guaranteed by its double pedicle with the two superficial temporal arteries. The scalp visor flap and its modifications have been used for the reconstruction of beards and moustaches in males after facial burns. Its use thus far, has been confined to resurfacing injured tissue. Only skin and subcutaneous tissue was injured in these patients [13,14].

**Figure 5:**
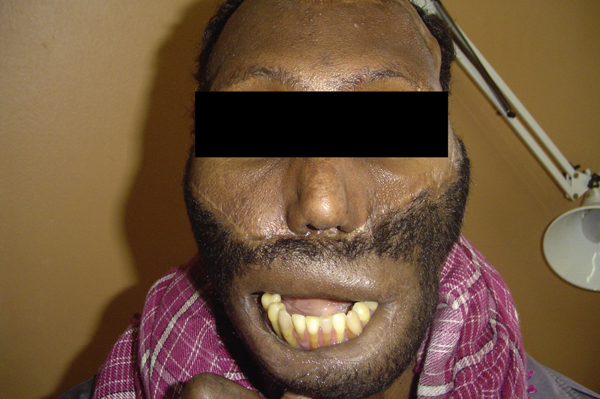
**Reconstruction showing neo-lips, moustache and beard at 1 year**.

Gousheh et al. used occipital scalp island flaps for total upper lip reconstruction [15]. There is no previous report of a simultaneous total upper and lower lip reconstruction, in a patient with a complete absence of lip tissue. The scalp visor flap in this patient was used to reconstruct the missing midfacial and lip tissue, as well as recreate a moustache and beard. Thus while simple to design and elevate, this flap served multiple functions, and gave invaluable solutions to otherwise technically difficult reconstructive needs in this patient. It gave an excellent color match with the remaining facial skin, provided aesthetic neo-lips, complete with a moustache and a beard, as a single unit (Figure 5). The patient and relatives found the reconstruction acceptable, but expressed a desire for a procedure that would allow oral continence. While a face transplant would give the best results in terms of aesthetics and function, this was not achievable in this patient and his environment at this time. An attempt at restoring oral continence using free functional muscle transfer will be made.

The major criticism against the classic visor flap is the defect left in the donor area - a large area of alopecia (Figure 4). This may be overcome by the use of a tissue expander, or, as in the present case, by acceptance by the patient, by virtue of existing cultural practice - the use of a headscarf, which is only taken off at night, or when bathing. Other donor defects, over the chest and neck, were more aesthetically acceptable, as well as being easily hidden by clothing (Figure 3).

Maintaining acceptable mouth opening and mandibular function post-reconstruction remains a significant problem [8], as in this patient, where at 2 years, the mouth opening remains at about 10 mm, down from an immediate postoperative 35 mm. Postoperative physiotherapy, with night splinting for up to a year, a highly patient- and guardian-dependent variable, is the main determinant of a good result.

## Conclusion

Jointly setting achievable goals with the patient and their relatives forms the foundation for successful noma reconstruction, as we found out. Most patients want to be able to eat sufficient amounts of food, be comfortable with their faces, and be accepted by the community within which they continue to live. While accommodating these basic expectations, goal setting must be realistic, achievable and acceptable to all the parties involved. The scalp visor flap is a simple yet excellent reconstructive solution for midfacial defects.

## Consent

Written informed consent was obtained from the patient for the publication of this paper and any accompanying images. A copy of the consent is available for review by the Editor-in-Chief of this journal.

## Competing interests

There is no association between the authors with any commercial firm, and no grants were given for this article. There are no competing interests in the publication of this article.

## Authors' contributions

This manuscript is original and has not been published previously nor is under review by any other journal. PN and LC are jointly responsible for the entire content of this article.
